# Voxel Based Morphometry Alterations in Mal de Debarquement Syndrome

**DOI:** 10.1371/journal.pone.0135021

**Published:** 2015-08-07

**Authors:** Yoon-Hee Cha, Shruthi Chakrapani

**Affiliations:** 1 Laureate Institute for Brain Research, Tulsa, Oklahoma, United States of America; 2 University of California Los Angeles, Los Angeles, California, United States of America; 3 Semel Institute for Neuroscience and Human Behavior, Los Angeles, California, United States of America; UCLA, UNITED STATES

## Abstract

**Background:**

Mal de debarquement syndrome (MdDS) is a disorder of chronic self-motion perception that occurs though entrainment to rhythmic background motion, such as from sea voyage, and involves the perception of low-frequency rocking that can last for months or years. The neural basis of this persistent sensory perception abnormality is not well understood.

**Methods:**

We investigated grey matter volume differences underlying persistent MdDS by performing voxel-based morphometry on whole brain and pre-specified ROIs in 28 individuals with MdDS and comparing them to 18 age, sex, and handedness matched controls.

**Results:**

MdDS participants exhibited greater grey matter volume in the left inferior parietal lobule, right inferior occipital gyrus (area V3v), right temporal pole, bilateral cerebellar hemispheric lobules VIII/IX and left lobule VIIa/VIIb. Grey matter volumes were lower in bilateral inferior frontal, orbitofrontal, pregenual anterior cingulate cortex (pgACC) and left superior medial gyri (t = 3.0, p<0.005_uncorr_). In ROI analyses, there were no volume differences in the middle occipital gyrus (region of V5/MT) or parietal operculum 2 (region of the parietoinsular vestibular cortex). Illness duration was positively related to grey matter volume in bilateral inferior frontal gyrus/anterior insula (IFG/AI), right posterior insula, superior parietal lobule, left middle occipital gyrus (V5/MT), bilateral postcentral gyrus, anterior cerebellum, and left cerebellar hemisphere and vermian lobule IX. In contrast, illness duration was negatively related to volume in pgACC, posterior middle cingulate gyrus (MCC), left middle frontal gyrus (dorsolateral prefrontal cortex-DLPFC), and right cerebellar hemispheric lobule VIIIb (t = 3.0, p<0.005_uncorr_). The most significant differences were decreased volume in the pgACC and increased volume in the left IFG/AI with longer illness duration (qFDR_corr_ <0.05). Concurrent medication use did not correlate with these findings or have a relationship with duration of illness. MdDS participants showed positive correlations between grey matter volume in pgACC and bilateral cerebellar lobules VIII/IX, which was not seen in controls.

**Conclusions:**

Individuals with MdDS show brain volume differences from healthy controls as well as duration of illness dependent volume changes in (a) visual-vestibular processing areas (IPL, SPL, V3, V5/MT), (b) default mode network structures (cerebellar IX, IPL, ACC), (c) salience network structures (ACC and IFG/AI) (d) somatosensory network structures (postcentral gyrus, MCC, anterior cerebellum, cerebellar lobule VIII), and (e) a structure within the central executive network (DLPFC). The identification of these associations may enhance future investigations into how exposure to oscillating environments can modulate brain function and affect motion perception as well cognitive and affective control.

## Introduction

Awareness of body position and agency of motion are fundamental not only to the production of movement but also to spatial cognition, memory, and emotive processes [1–3]. Understanding which brain networks are involved in driving a false sense of body motion may illuminate the anatomical connections that underlie many disorders of spatial perception and postural control that have cognitive and affective features [4–6].

A useful model to study brain structures involved in abnormal self-motion perception is a disorder termed *mal de debarquement syndrome* (MdDS). MdDS is a disorder of intractable perception of self-motion that occurs after prolonged exposure to oscillating environments such as occurs during sea travel [7]. Unlike common forms of landsickness, the rocking and swaying sensations that occur in MdDS can last for months or years leading to significant morbidity [8]. There is currently no explanation for why the common experience of post-motion rocking dizziness does not go away in some individuals. Relevant clinical features are that MdDS typically affects middle-aged individuals and is more common in women [7]. People experiencing MdDS represent a unique subject group in which to study brain volume changes associated with motion perception because their symptoms are chronic, they occur in the absence of central or peripheral vestibular injury, and the affected individuals are otherwise physically healthy [7,9,10].

Voxel based morphometry (VBM) is a technique used to make voxel-by-voxel comparisons of segmented grey matter volume or concentration differences and can be useful in showing brain volume changes in chronic illnesses that are not evident in clinical scans [11]. Brain volume changes secondary to peripheral vestibular nerve injury such as occurs in vestibular neuritis or acoustic neuroma resection have been shown to occur in motion sensitive area V5/MT, inferior parietal lobule, posterior insula, and posterior hippocampus [12,13]. Though these examples of central compensation after peripheral injury are helpful starting points in understanding where perception of body motion is processed, they may be difficult to study to understand chronic ongoing motion illusions because of their associated hearing and eye movement abnormalities, continuously changing central compensatory processes and the limited duration of the motion illusion.

One approach to identifying which neural substrates might be affected in the process of motion entrainment that leads to MdDS is to examine brain volume changes. Here, VBM was used to identify structural changes in grey matter to better delineate the neural basis of MdDS. An ancillary question addressed in this investigation was whether grey matter volume in specific areas correlated with a longer history of MdDS. We further determined whether brain volumes within the identified structures correlated differently in MdDS and if there were any associations with the kinds of medications that are used to treat MdDS.

## Materials and Methods

### Subject Recruitment

The Institutional Review Board of the University of California Los Angeles, specifically Medical IRB 3, which oversees neuroscience research, approved all study procedures, which were performed according to the Declaration of Helsinki guidelines. Participants provided written informed consent.

Participants with a history of unambiguous motion-triggered symptoms of MdDS were recruited by advertisement through the MdDS Balance Disorders Foundation website (www.mddsfoundation.org) or through a database of patients diagnosed with MdDS at the investigator’s institution. Inclusion into the study required: 1) a typical history of chronic rocking dizziness occurring within two days of disembarking from a moving vessel such as from sea, air, or land-based travel; and 2) symptoms lasting at least three months without any other cause found after evaluation by a neurologist or otolaryngologist. Participants without a history of clear motion triggered rocking dizziness or a history that was otherwise suggestive of peripheral vestibular nerve injury were excluded. Healthy control participants were selected based on a negative history for any form of dizziness or imbalance. They were proportionately matched for age, sex, and handedness. All participants were required to be normotensive (blood pressure <140/90 and pulse 60–100 beats per minute) and to pass a screening neurological examination. Because of hemispheric structural asymmetry driven by handedness, only right-handed participants were included. All participants were scanned on the same MRI scanner between June 2009 and November 2012 on the same protocol.

### MRI Imaging

#### Structural scans

Participants were scanned on a Siemens Magnetom Trio 3Tesla scanner with a 12-channel head coil at the Ahmanson-Lovelace Brain Mapping Center (Los Angeles, CA.). Specifications for Magnetization Prepared Rapid Acquisition Gradient Echo imaging were as follows: 192 slices at 1mm slice thickness, voxel size: 1.0mmx1.0mmx1.0mm, field of view: 256mm, flip angle: 9 degrees, repetition time (TR) = 1900ms, echo time (TE) = 3.25ms.

### VBM Analysis

Because of normalization artifacts from whole brain volume processing that can disproportionately affect the cerebellum, we separately analyzed cerebral versus cerebellar structures.

#### Cerebral VBM

Cerebral VBM was performed using the VBM8 toolbox in SPM8 (http://www.fil.ion.ucl.ac.uk/spm) with Matlab 2009b (Mathworks, Natick, MA.). Structural images were reoriented using SPM8 to align the images along the anterior-commissure (AC) and posterior-commissure (PC) planes. Images were reoriented to place the origin (0,0,0) at the AC. Using the Matlab based VBM8 toolbox each image was segmented into grey matter, white matter and CSF volumes using the tissue probability map within the segmentation toolbox in SPM8. Only grey matter images were used for these analyses. High dimensional spatial normalization using the DARTEL MNI 152 template was performed with 0.15 MRF weighting. Modulated images using only non-linear normalization in order to determine grey matter volume corrected for individual brain sizes were created. A brain mask removing the cerebellum was created with the WFU Pick Atlas version 3.0.4 [14,15]. An absolute threshold masking of 0.1 was used for grey matter assignment to avoid edge effects between grey and white matter. Images were smoothed with an isotropic Gaussian kernel with at 8mm FWHM. This is a medium level of smoothing for a cerebral VBM study (ranges 4–12mm) that mitigates the subject-to-subject variations in registration to the normal template. A sample homogeneity test was performed to remove any outliers that were above or below two standard deviations of the mean brain volume.

#### Cerebellar VBM

We used the spatially unbiased atlas template of the cerebellum and brainstem (SUIT) version 2.7 in order to precisely localize foci within the cerebellum [16,17]. SUIT uses a higher resolution template of the cerebellum for normalization than the standard SPM T1 template and provides localization into 28 distinct cerebellar regions. Whole brain images previously aligned along the AC-PC axis were subjected to the isolation procedure under SUIT, which separates the cerebrum from the cerebellum based on cortical grey matter thickness. Visual inspection confirmed high quality isolation. Normalization with DARTEL to the standard SUIT template was performed followed by re-slicing into 1x1x1mm voxels and smoothing with a 4mm FWHM isotropic kernel. A smaller smoothing kernel was used for the cerebellum than the cerebrum since cerebellar architecture is more homogeneous than the cerebrum. Modulated images corrected for individual brain volumes were used.

#### Statistical Analyses

Whole brain voxel-by-voxel comparison between MdDS participants and controls was performed with an unpaired two-sample t-test assuming independent samples and unequal variances thresholded at a t value of 3.0 and extent of 30 voxels in the cerebrum and 10 voxels in the cerebellum. These were the effect sizes that we wished to see. For contrast images, this translates to a *p* value <0.005 with 40 degrees of freedom. For the multiple regression analysis, this translates to a *p* value <0.005 with 21 degrees of freedom. We did not correct for whole brain voxel number since many of the areas in our results have been shown to be in functionally related regions and we would have risked overcorrecting the data. Covariates included age and total grey matter volume. Because of an initial finding of prefrontal volume decrements in MdDS, we also added anxiety (HADA) and depression (HADD) subscores of the Hospital Anxiety and Depression Scale (HADS) as additional covariates in our final analysis to control for volume differences that could be due to depression or anxiety [18]. In order to determine whether brain volume changes correlated with length of illness, we performed a multiple regression analysis using duration of illness in months.

Since brain volume changes could be related to the use of medications, we determined which medications were used differently between MdDS participants and controls. We then performed an ANOVA using the number of medications found to be different between the two groups as the number of factors with each factor having two levels.

Peak voxels from the contrast images between MdDS vs Controls as well as those determined by regression for duration are presented in Montreal Neurologic Institute coordinates with localization determined by the Jülich atlas version 2.0 [19]. Anatomical labels are provided for all foci and probabilistic functional labels are provided in parentheses, when available. Mean volume in clusters with a corrected false detection rate (FDR) significance of p<0.05 were extracted to determine whether there were correlations with duration of illness or the use of certain medications.

#### V5/MT and OP2 ROIs

ROIs were created for areas V5/MT (human extrastriate region hOC5) and OP2 (the human parietal operculum region 2, the anatomical center of the parietoinsular vestibular cortex) according to the Jülich probabilistic atlas [19–22]. Mean volumes were extracted using the MarsBaR tool [23].

## Results

Twenty-nine right-handed individuals (24 female) with MdDS (43.0+/-10.2 years) and 18 healthy volunteers (14 female) (43.9+/-12.4 years) participated in this study (*p* = 0.788 for the difference). Of the MdDS participants, 16 developed symptoms after sea travel (usually from a cruise), 10 after air travel, and three after land-based travel (car or train). Duration of symptoms ranged from 3–240 months. If the outlier of 240 months was excluded, the mean duration was 35.7+/-28.7 months with a median of 24 months. All but one subject had both brain MRIs and vestibular function testing available through previous clinical evaluations, which were normal in all cases. These evaluations came as part of their medical records and source data (e.g. actual tracings) were not available. MdDS individuals had higher ratings of anxiety (HADA 8.35+/-4.19) and depression (HADD 6.43+/-3.95) than healthy comparison participants (HADA 3.83+/-3.73, p<0.001, HADD 1.22+/-1.40, p<0.001).

There were no major central lesions such as evidence of prior ischemia or tumors in any of the images acquired as part of this study. After a sample homogeneity test on the VBM data, one scan from the MdDS group was identified as a significant outlier, given a total grey matter volume exceeding two standard deviations from the mean. This sample, from a female subject, was removed from the analysis. Subsequent analyses on contrast images were performed on 28 MdDS and 18 Control participants. The participant with symptoms lasting 240 months was removed from the regression analysis for duration since this was over three standard deviations from the mean for duration and would have created too much leverage effect.

### MdDS and Control contrasts

**Fig 1:**
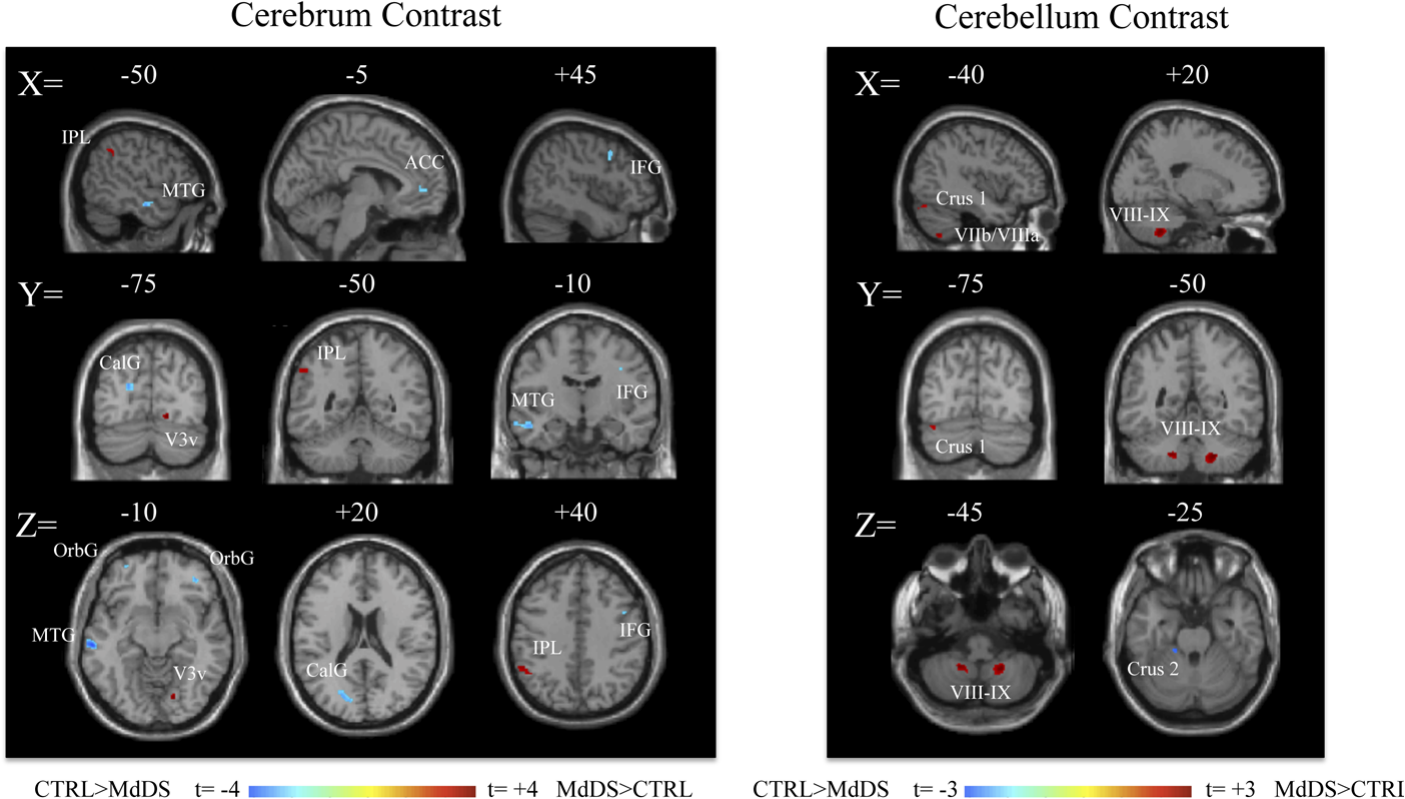
Contrast images of grey matter volume differences between MdDS and controls shown at a threshold of t = 3.0, extent voxels 30 for cerebrum and 10 for cerebellum. Blue scales images represent areas with higher volume in Controls; red areas represent higher volumes in MdDS participants. Scale values are in t score. Coordinates are in MNI space.

Relative increases in brain volume were seen in the MdDS participants in the left inferior parietal lobule (IPL), right ventral occipital lobe, and right temporal lobe. Relative decreases were seen primarily in orbital and frontal regions, most notably in the pregenual region of the anterior cingulate cortex (pgACC) (**Fig 1, Table 1**). Grey matter was relatively increased in the caudal cerebellum in hemispheric lobules VIIIb and IX bilaterally as well as in left Crus I, VIIa and VIIIa in MdDS participants while a relative decrease was only seen in a small portion of left cerebellar Crus II (**Fig 1, Table 2**).

**Table 1:** Cerebral Contrasts.

**MdDS>CTRL**						
	**T**	**ke**	**X**	**Y**	**Z**	**Maximum probability**
**Cluster 1**	3.37	73	-52	-46	39	L Inferior Parietal Lobule
**Cluster 2**	3.96	46	16	-76	-9	R Inferior Occipital Gyrus (V3v)
**Cluster 3**	3.48	31	51	20	-24	R Temporal Pole
**CTRL>MdDS**	** **	** **	** **	** **	** **	** **
	**T**	**ke**	**X**	**Y**	**Z**	**Maximum probability**
**Cluster 1**	4.38	426	-60	-24	-11	L Middle Temporal Gyrus
**Cluster 2**	3.78	141	-20	-75	21	L Calcarine Gyrus
**Cluster 3**	3.98	128	-28	56	-6	L Middle Orbital Gyrus
**Cluster 4**	3.48	107	40	-4	34	R Inferior Frontal Gyrus, p. opercularis
**Cluster 5**	3.78	60	39	44	-12	R Middle Orbital Gyrus
**Cluster 6**	3.29	49	-6	44	3	L Anterior Cingulate Cortex

**Table 2:** Cerebellar Contrasts.

**MdDS>CTRL**						
	**T**	**ke**	**X**	**Y**	**Z**	**Maximum probability**
**Cluster 1**	3.89	817	22	-50	-44	R Cerebellum, VIIIb and IX
**Cluster 2**	3.77	313	-19	-49	-44	L Cerebellum, VIIIb and IX
**Cluster 3**	3.62	240	-39	-59	-53	L Cerebellum, VIIb and VIIIa
**Cluster 4**	3.40	156	-38	-78	-20	L Cerebellum, VIIa Crus 1
**CTRL>MdDS**						
	**T**	**ke**	**X**	**Y**	**Z**	**Maximum probability**
**Cluster 1**	3.24	13	-20	-83	-28	L Cerebellum, VIIa Crus 2

### Multiple regression for duration

**Fig 2:**
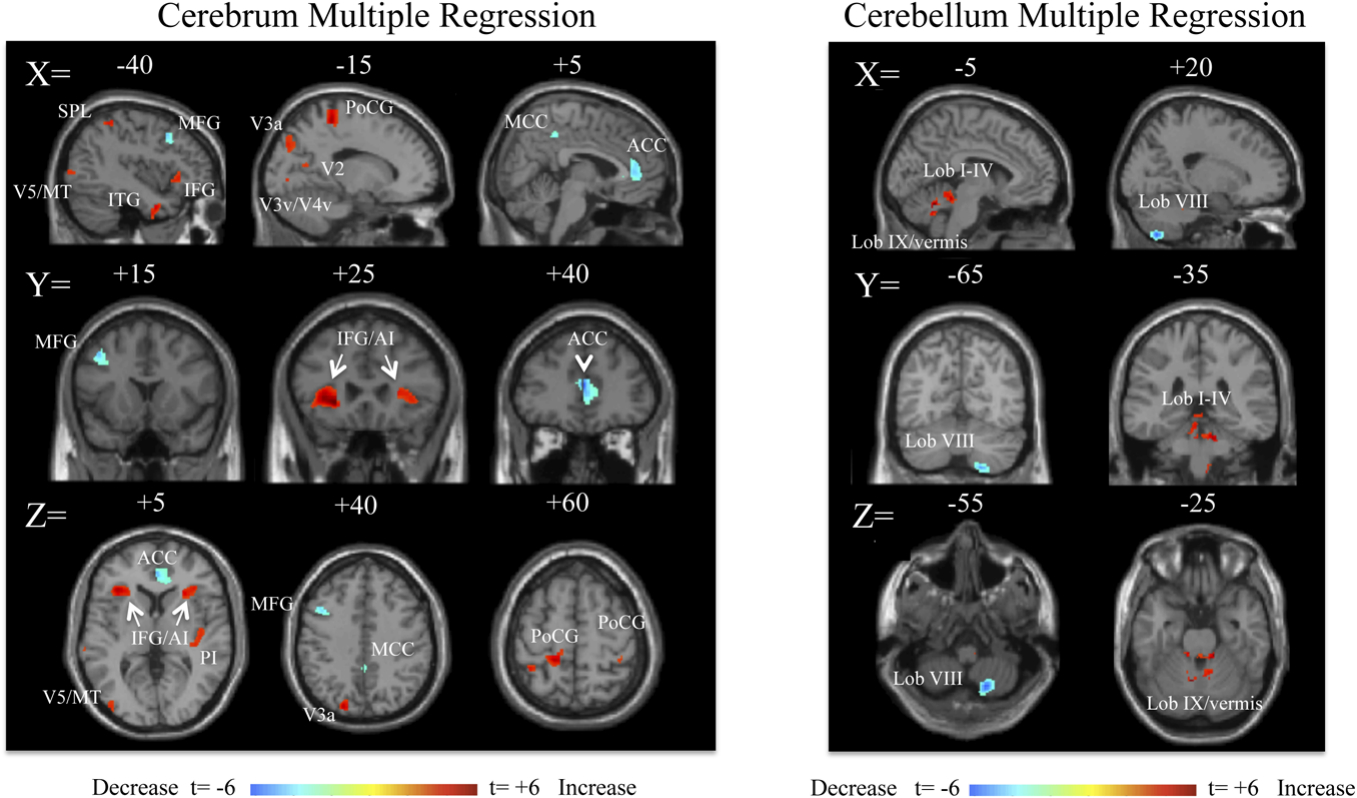
Multiple regression analysis for duration in months, shown at a threshold of t = 3.0, extent voxels 30 for cerebrum and 10 for cerebellum for 27 MdDS participants. Blue areas represent areas of lower volume in individuals with longer duration of illness; red areas represent areas of higher volume in individuals with longer duration of illness. Scale values are in t scores. Coordinates are in MNI space.

Twenty-seven participants were entered into a multiple regression analysis to determine which brain regions varied in volume with respect to duration. As a function of duration, grey matter volume increased in bilateral inferior frontal gyrus/anterior insular (IFG/AI) cortex, right posterior insula, superior occipital gyrus region (V3a), postcentral gyrus (somatosensory cortex), left middle occipital gyrus (V5/MT), superior parietal lobule (SPL), and the left temporal lobe (**Fig 2, Table 3**). In the cerebellum, grey matter volume increased primarily in the anterior (lobules I-IV) cerebellum, left hemispheric lobule IX, and vermian lobule IX (**Fig 2, Table 3**). Cerebral volume in the pgACC, posterior middle ACC, left middle frontal gyrus (region of dorsolateral prefrontal cortex, DLPFC), and, the right cerebellar lobule VIIIa/b decreased as a function of duration (**Fig 2, Table 4**). The left IFG/AI and the ACC volume changes surpassed a whole brain corrected FDR p<0.05 at the cluster level. Age showed no correlation with duration (R^2^ = 8.5x10^-5^). ROI analyses in areas V5/MT and OP2 did not reveal any differences in mean volume across the clusters: MdDS Left V5 = 0.433, CTRL Left V5 = 0.418, MdDS Right V5 = 0.430, CTRL Right V5 = 0.447, MdDS Left OP2 = 0.363, CTRL Left OP2 = 0.366, MdDS Right OP2 = 0.402, CTRL Right OP2 = 0.389, all p>0.2.

**Table 3:** Multiple Regression: Positive Correlation with Duration.

**Cerebrum**	** **	** **	** **	** **	** **	** **
	**T**	**ke**	**X**	**Y**	**Z**	**Maximum probability**
**Cluster 1**	6.29	947	-26	30	-3	L Inferior Frontal Gyrus, p. orbitalis
**Cluster 2**	5.10	709	-15	-37	61	L Postcentral Gyrus
**Cluster 3**	5.60	384	-18	-81	36	L Superior Occipital Gyrus (V3a)
**Cluster 4**	4.08	334	27	24	4	R Inferior Frontal Gyrus, p. orbitalis
**Cluster 5**	3.93	302	40	-21	7	R Heschls Gyrus/ Insula Ig1/Ig2
**Cluster 6**	4.87	279	26	-72	19	R Cuneus
**Cluster 7**	4.07	241	-51	-12	-26	L Inferior Temporal Gyrus
**Cluster 8**	4.65	197	-56	-21	0	L Middle Temporal Gyrus
**Cluster 9**	4.52	187	51	-25	54	R Postcentral Gyrus
**Cluster 10**	4.13	174	-40	-45	57	L Superior Parietal Lobule
**Cluster 11**	4.26	135	-46	-4	-15	L Middle Temporal Gyrus
**Cluster 12**	4.34	109	-40	2	-33	L Inferior Temporal Gyrus
**Cluster 13**	3.55	100	21	-58	10	R Calcarine Gyrus (V1/V2)
**Cluster 14**	3.53	53	-42	-85	4	L Middle Occipital Gyrus (V5/MT)
**Cluster 15**	3.45	42	-33	-9	-23	L Amygdala (lateral basal)
**Cluster 16**	3.91	38	-46	12	12	L Inferior Frontal Gyrus, p. triangularis
**Cluster 17**	3.58	38	-15	-82	-2	L Lingual Gyrus (V3v/V4v)
**Cluster 18**	3.28	35	-16	-63	13	L Calcarine Gyrus (V2)
**Cluster 19**	3.51	31	46	0	-11	R Temporal Pole
**Cerebellum**						
	**T**	**ke**	**X**	**Y**	**Z**	**Maximum probability**
**Cluster 1**	4.52	108	-6	-38	-17	L Lobule I IV, hemisphere
**Cluster 2**	5.03	49	8	-50	-27	R Lobule I-IV, hemisphere
**Cluster 3**	5.96	44	-6	-56	-23	Not assigned, hemisphere lobule V likelihood 1%
**Cluster 4**	6.03	41	14	-34	-27	R Lobule I-IV, hemisphere
**Cluster 5**	4.35	13	-6	-58	-31	L Lobule IX/Vermis IX
**Cluster 6**	4.42	12	8	-38	-59	Not assigned by atlas, probable medulla

**Table 4:** Multiple Regression: Negative Correlation with Duration.

**Cerebrum**						
	**T**	**ke**	**X**	**Y**	**Z**	**Maximum probability**
**Cluster 1**	6.40	640	2	40	13	R Anterior Cingulate Cortex
**Cluster 2**	4.83	221	-42	15	43	L Middle Frontal Gyrus
**Cluster 3**	3.98	105	3	-40	45	R and L Middle Cingulate Cortex
**Cluster 4**	5.16	39	-2	40	13	L Anterior Cingulate Cortex
**Cerebellum**						
	**T**	**ke**	**X**	**Y**	**Z**	**Maximum probability**
**Cluster 1**	6.24	165	18	-66	-55	R Lobule VIIIb/VIIIa, hemisphere

**Fig 3:**
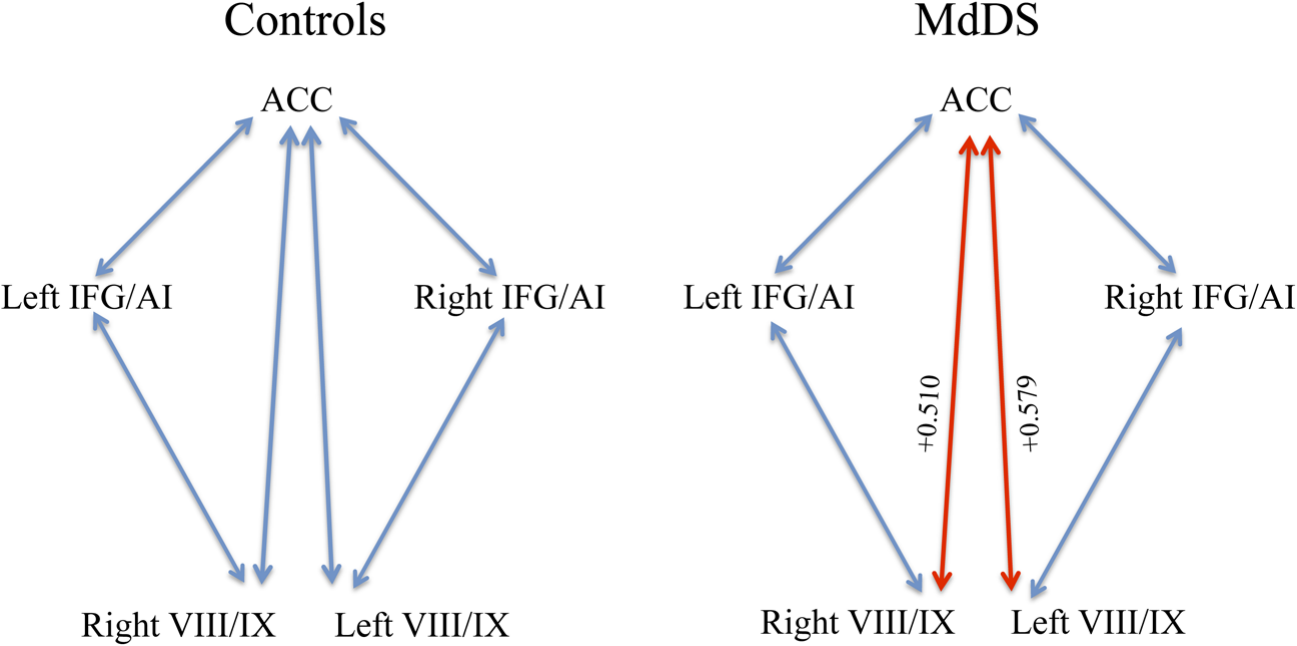
Pearson’s correlation coefficients between nodes with the most significant volume changes with time. Blue lines signify relationships that are not significant. The red lines are correlations at <0.05.

Mean volumes in areas of the most significant differences were extracted to determine the correlation coefficients relative to each other as well as to duration: pgACC, right and left IFG/AI, and bilateral cerebellar VIII/IX. Notably pgACC volume was negatively correlated with duration with a correlation coefficient of -0.633, p<0.05 (corrected), indicating a moderately strong negative correlation. Both left and right IFG/AI volumes were positively correlated with duration with correlation coefficients of +0.440 and +0.427, respectively, p<0.05 (corrected). There was a high positive correlation between ACC and bilateral cerebellar VIII/IX volume in MdDS participants because both volumes decreased with duration. **Fig 3**shows the differences in volume correlations in MdDS relative to controls for correlations significant to p<0.05. All other correlations were not significant after Bonferroni correction for multiple comparisons.

The use of the following major classes of medications was assessed: selective serotonin reuptake inhibitors (SSRIs), tricyclic amines, benzodiazepines, anticonvulsants, triptans, beta blockers, calcium channel blockers, ACE inhibitors and angiotensin blockers, hormone replacement, thyroid replacement, vitamins, and statins. Only the use of SSRIs (p<0.05) and benzodiazepines (p<0.001) were significantly different between the two groups. This is expected because these are the main classes of medications that have been shown to be symptomatically effective for MdDS [10]. A 2x2 ANOVA with SSRI and benzodiazepines (factor = drug class, level = present or not present) was performed for duration, ACC, and left IFG/AI volumes. There was no significant effect of the use of these medications either on the duration of illness or the volumes of these areas (**Table 5**).

**Table 5:** Association between SSRI and benzodiazepine use with duration and volume changes.

	Duration		ACC		Left IFG/AI	
F (2,27)	F-ratio	*p* value	F-ratio	*p* value	F-ratio	*p* value
SSRI	0.022	0.884	0.198	0.660	0.103	0.751
Benzodiazepines	0.799	0.381	1.324	0.262	2.462	0.130
SSRI x Benzodiazepines	3.711	0.067	0.004	0.951	1.230	0.279

## Discussion

This study shows that there are grey matter volume differences in brain areas involved in processing somatosensory and spatial information as well as those that mediate awareness of interoception and attentional control in individuals with MdDS. The general pattern of primary brain volume increases seen were in sensory association areas such as IPL and SPL and functional areas V5/MT and area V3v as well as cerebellar lobes VIII and IX which are related to the somatosensory network and the default networks, respectively. As a function of duration of illness, grey matter volume significantly decreased in ACC while increasing in bilateral IFG/AI, important hubs of the salience network involved mediating interoceptive awareness and in regulating limbic activity [24].

### Role of the anterior cingulate cortex

The ACC is composed of a dorsal division that is functionally related to the evaluation and response to stimuli with rich connectivity to lateral prefrontal cortex whereas the ventral ACC functions to regulate limbic activity through its connections to the amygdala, periaqueductal grey, and the hypothalamus [25,26]. In our study, the specific region of the ACC with differential volume was the pregrenual ACC (pgACC), which is functionally considered part of the ventral-rostral group. The pgACC plays a role in emotional conflict regulation, the extinction of conditioned fear, and in the planning of responses to future threats; activations have been associated with decreased amygdala responses [27–30].

### Role of the inferior frontal gyrus/anterior insular cortex

Paired with the decrease in pgACC volume with duration of illness was an increase in volume of a frontoinsular area that included both the inferior frontal gyrus and the anterior insula (IFG/AI). Objective sensory percepts such as of hot and cold stimuli are processed in the posterior insula whereas subjective awareness and evaluation of those stimuli are mediated in the anterior insula (AI) [31,32]. It is hypothesized that the AI harbors a representation of the feelings associated with body movements. A model has been proposed for a posterior-to-anterior pathway of sensory integration that progressively incorporates environmental, emotional, and cognitive factors until a global percept of an experience is finally developed in the AI [33].

The ACC, along with the AI, amygdala, and the hypothalamus are part of the “salience” network, which serves to reorient attention to functionally relevant internal and external stimuli [34]. ACC and AI along with DLPFC and posterior parietal cortex are also part of the cognitive control network [35]. Our data showed duration related volume decreases in the left middle frontal gyrus, the region of the DLPFC, indicating that both a cognitive network and an emotional regulation network are affected in MdDS. The decrease in left DLPFC volume with increasing duration is consistent with our previous finding that excitatory repetitive transcranial magnetic stimulation (rTMS) over the left DLFPC can acutely decrease the intensity of the rocking perception in MdDS but that the likelihood of response to rTMS decreases as a function of duration of illness [36]. Hubs that are common to multiple networks may be points through which emotional and cognitive information can influence each other. The volume differences found in this study of two such important hubs may be relevant to why patients with MdDS often note severe cognitive and affective control problems associated with the motion perceptions [37].

Studies of emotional awareness typically show concurrent activation of ACC and AI, whether the emotion is positive or negative but there are notable instances when ACC and AI activations are dissociated. In particular, tasks requiring time estimation, attention to rhythm, and body movements that do not involve agency i.e. the sense that one is generating the movement, are associated with activation of AI without activation of ACC [38–40]. These differences may be pertinent to MdDS since ACC and IFG/AI volume showed an inverse relationship relative to duration in our data and the trigger that causes MdDS is one of rhythmic motion. The caveat to this interpretation, however is that brain activity and volume are not necessarily correlated.

### Role of the cerebellum

Cerebellar volume changes in hemispheric lobes VIIIa/b and IX (along with vermian lobule IX to which it projects) in these data are consistent with the cerebral volume changes that were seen in bilateral primary sensory cortex and ACC. Cerebellar lobule VIII is considered part of the secondary motor representation within the cerebellum being functionally connected to premotor cortex; the primary motor representation is within lobules I-IV, which are connected to primary motor cortex [41]. Cerebellar lobule IX has strong functional connectivity with the default mode network [41,42]. Our regression analysis showed increased volume in the anterior cerebellum with duration but was contrasted by decreased volume in the right cerebellar VIIIa/b. One factor explaining this difference might be that the right lobule VIII has the distinction of being the only lobule within the cerebellum to have intrinsic activity negatively correlated with every section of the precuneus, a region that has rich connections to somatomotor cortex and hubs within the default mode network; the precuneus is involved in visuospatial processing, attention, and memory [43,44]. Therefore, lobule VIII may have additional influence on cerebral networks outside of its role in the motor system perhaps by its influence on the default mode network. This interpretation would be consistent with the increased volume also seen in lobules IX. Volumes in cerebellar lobules VIII/IX and ACC were positively correlated in MdDS participants but not in Controls though overall VIII/IX volume was higher and ACC volume was lower in MdDS subjects. The most straightforward explanation for this discrepancy would be that VIII/IX maybe higher in individuals with MdDS at baseline before the onset of their disorder (perhaps a risk factor) but that volume decreases with time in parallel with a decrease in ACC volume, perhaps as a compensatory process. Only a longitudinal study could verify this possibility.

The cerebellum is situated to make important contributions to major resting state brain networks. Converging sensory input into the cerebellum allows it to be part of the process of making predictions about future sensory experiences, allowing predictable future events to be cancelled out in order to highlight unpredictable functionally relevant information [45]. This process is particularly important when in an oscillating environment. A model for rhythmic sensory input needs to be made in order to release repetitive postural adjustment strategies from conscious perception or to make an efference copy that cancels out the afferent input in order to decrease attention to repetitive sensory stimuli [46]. This process is particularly relevant during sea travel in which the development of ‘sea legs’ (adaptation to the sea) is associated with reduction in motion sickness. Our data shows that areas that are functionally connected to somatomotor regions, motor planning, and default mode networks within the cerebellum show volume changes in MdDS, a disorder in which feelings of motion occur at rest. Part of the adaptation to background oscillating motion may be to redefine a new baseline and the neural substrate for that may be alterations in the default network.

### Role of area V5/MT

Though we did not detect any primary differences in brain volume in motion sensitive area V5/MT either through whole brain interrogation or ROI analysis, the left V5/MT in the superior occipital gyrus did emerge as an area that increases in brain volume with increased duration of illness. We would therefore submit that it is not the primary driver of the motion perception but may become a more important contributor with time. Apparent motion elicited by illusory contours, imagined motion, and triggering illusions of self-motion can all activate V5/MT [47,48]. The enhanced functional connectivity between V5/MT and the entorhinal cortex (EC) found in our previous study suggests that there may be enhanced transfer of motion information to the EC from V5/MT or that motion information from V5/MT can more efficiently drive EC activity with time in MdDS [49].

### Role of the parieto-insular vestibular cortex and other areas

We did not find volume differences in the parieto-insular vestibular cortex (PIVC), located in the parietal operculum (OP2). This area is considered to be the best candidate for a primary vestibular cortex, though clearly it is not a unimodal area [50,51]. The vestibular system has a widespread cortical representation, however and stimulation of multiple brain regions can trigger feelings of motion. Perceptions of non-spinning self-motion such as linear translation and oscillating motion (e.g. rocking and swaying) can be elicited by electrically stimulating the ACC and functionally connected areas like the precuneus and the frontal operculum [52,53]. In contrast, perceptions of rotation or tilting are more likely to be elicited by stimulating the posterior temporal lobe [53]. Therefore, our finding of altered volume in the pgACC and frontal opercular area (IFG) is more consistent with direct stimulation studies that elicit sensations of rocking motion rather than rotational vertigo. This may help explain why chronic rocking dizziness specifically is often associated with anxiety and affective disorders [4,5]. This was our rationale for using the HADS scores to regress out specific brain volume trends associated with high mood or anxiety scores.

### Limitations

There are several limitations to our study. Brain volume differences may be due as much to compensatory processes as they may be direct drivers of the symptoms so we do not know whether the volume changes observed are a cause or an effect of the motion sensation; we have taken a snapshot of the affected individuals’ brains at only one time point. A within-subject longitudinal study in which symptoms can be varied (such as with therapy) would be better positioned to determine the direction of brain volume changes as they pertain to symptom changes.

Second, our basic contrast between MdDS and Controls were presented with uncorrected p-values and it can be argued that many of the voxels seen in the contrasts could be seen by chance. Only the pgACC and the left IFG/AI were significantly increased with duration of illness at a corrected qFDR of p<0.05. The finding of ACC differences between MdDS and Controls along with volume reductions as a function of duration suggests that this area is a relevant structure in the pathology of MdDS, despite not reaching statistical threshold in the baseline contrast images. Moreover, the volume increases in the caudal cerebellum (VIII/IX) were symmetrically increased in MdDS, making it less likely they were due to random chance. As the first study of its kind in terms of showing differences in brain volume associated with a chronic sensation of motion in a non-injury model, we prioritized showing more data than less in order to enhance future efforts to finding common pathways in disorders of abnormal sensory perceptions. We could have limited our area of analysis but based on prior studies that have shown a very wide distribution of vestibular projections and the large number of brain areas that, when stimulated, generate feelings of body motion, restricting the analysis to a limited number of ROIs and performing small volume correction was going to risk missing the identification of potentially significant brain areas.

Third, although we controlled for age, sex, and handedness, there were many other variables that we could not control such as how much lifetime motion exposure each group had experienced, e.g., the number of cruises or plane flights people with and without MdDS underwent. The latter would constitute an almost infinite number of permutations of type of travel, distance, vessel, and age at exposure and was not practical for these analyses. Even if these data were ascertained, they would have been unverifiable.

Therefore, fourth, we only used duration for our regression analysis because it was the only truly reliable input variable, as opposed to severity or level of disability, as those are far too subjective. Factors related to duration are the most important to understanding why the symptom of rocking dizziness after motion exposure has a tendency to persist the longer it lasts [10].

Finally, we did not functionally define areas V5/MT or PIVC, which could be different between individuals and thus cancel out each other’s signals when analyzed as a group. Instead, we used maps of these regions that were carefully delineated in a well-established atlas. We only performed ROI analyses for the V5/MT and the PIVC since these are areas of exceptional interest for understanding motion perception and have been well-defined in the Jülich atlas. Future brain volume studies in MdDS may limit the analysis to specific brain areas also identified in the present study, however.

Despite these caveats, the identification of major hubs of the saliency and default mode networks as being altered in MdDS is consistent with some of the clinical features observed such as heightened sensitivity to environmental stimuli and the rocking dizziness predominantly being experienced when the individual is at rest but becoming nulled with re-exposure to passive motion [10,54]. Volume reductions in prefrontal areas, particularly DLFPC, for example are consistent with problems with attention. MdDS as a disease model may thus be useful in understanding pathways involved in motion adaptation and ultimately in illuminating the association between disorders of self-motion perception and cognitive and affective symptoms. Identification of the relevant networks is important in deriving a more complete understanding of the process of body motion awareness and ultimately in identifying pathways through which to intervene for therapeutic purposes.
